# Can We Omit Systematic Biopsies in Men with a Positive MRI During Repeat Biopsies for Active Surveillance for Prostate Cancer? Results from the PRIAS Study

**DOI:** 10.1016/j.euros.2026.09.006

**Published:** 2026-10-13

**Authors:** Jeroen J. Lodder, Sebastiaan Remmers, Markus Graefen, Francisco Lozano-Uruñuela, Cristina Marenghi, Philipp Papavassilis, Antti Rannikko, Diederik Somford, Frederic Staerman, Mikio Sugimoto, Karl H. Tully, Roderick C.N. van den Bergh, Monique J. Roobol, the Prostate Cancer Research International: Active Surveillance (PRIAS) consortium

**Affiliations:** a Department of Urology, Erasmus MC Cancer Institute, Erasmus University Medical Center, Rotterdam, The Netherlands; b Martini-Klinik Prostate Cancer Center, University Hospital Hamburg-Eppendorf, Hamburg, Germany; c Department of Urology, Hospital Universitario de Navarra, Pamplona, Spain; d Department of Medical Oncology, Fondazione IRCCS Istituto Nazionale dei Tumori, Milan, Italy; e Department of Urology, University Hospital Muenster, Muenster, Germany; f Department of Urology, Helsinki University Hospital, Helsinki, Finland; g Department of Urology, Canisius Wilhelmina Hospital, Nijmegen, The Netherlands; h Department of Urology, Polyclinique Reims-Bezannes, Bezannes, France; i Department of Urology, Kagawa University, Kagawa, Japan; j Department of Urology and Neurology, Marien Hospital Herne, Herne, Germany; k Department of Urology, Sint Franciscus Hospital, Rotterdam, The Netherlands

**Keywords:** Active surveillance, De-escalation, Diagnostics, Prostate cancer, Repeat biopsy

## Abstract

**Background and objective:**

This study evaluated the added value of systematic biopsies (SBx) in addition to targeted biopsies (TBx) for detecting upgrading during repeat biopsies on active surveillance (AS) using data from the Prostate Cancer Research International Active Surveillance study.

**Methods:**

We analyzed repeat biopsies of men who underwent magnetic resonance imaging (MRI) and SBx at diagnosis, with or without TBx depending on the presence of an MRI lesion, had at least one positive (Prostate Imaging Reporting and Data System 3–5) follow-up MRI, and received both SBx + TBx at subsequent repeat biopsy. Upgrading compared with diagnostic biopsy was assessed for TBx only, SBx only, and both, using three definitions: any upgrading, upgrading to grade group (GG) ≥2 with cribriform growth or intraductal carcinoma (≥GG2+), and upgrading to ≥GG3. Cumulative incidences were calculated using each detection method as competing events. For upgrading ≥GG2+ detected by SBx only, the laterality relative to the MRI lesion was determined.

**Key findings and limitations:**

We analyzed 650 repeat biopsies in 568 patients with a median follow-up of 3.0 yr (interquartile range = 1.8–4.3) for men still on AS. During available follow-up, any upgrading occurred in 238 biopsies (37%), of which 47 (7.2%) were detected by SBx only. Upgrading to ≥GG2+ and ≥GG3 was observed in 100 biopsies (16%) and 78 biopsies (12%), respectively, with SBx alone identifying 19 (3.0%) and 15 (2.3%) of these patients. At 6 yr, the cumulative incidence of ≥GG2+ upgrading was 27% (95% confidence interval [CI] = 21–32%), with 5.4% (95% CI = 2.9–9.1%) for upgrading detected exclusively by SBx. Three of the 19 cases with upgrading to ≥GG2+ detected by SBx only were located contralateral to the MRI lesion (0.5% of all biopsies). Limitations include the retrospective design, which may introduce selection and registration biases.

**Conclusions and clinical implications:**

In men on AS who underwent diagnostic MRI with SBx (with or without TBx) and a positive follow-up MRI, SBx has limited added value during repeat biopsies, particularly for contralateral sampling, supporting a targeted and perilesional biopsy approach.

## Take Home Message

Systematic biopsies add limited diagnostic value during repeat biopsy in men on active surveillance with positive follow-up magnetic resonance imaging. Targeted and ipsilateral systematic biopsies detect nearly all upgrading cases, supporting a targeted and perilesional biopsy approach in this setting.

ADVANCING PRACTICE **What does this study add?**

This study provides real-world evidence on the added value of systematic biopsies during repeat biopsy in men on active surveillance with a positive follow-up magnetic resonance imaging (MRI). Using data from a large, international, multicenter cohort, it captures variability in MRI quality, radiologist expertise, and biopsy techniques outside expert centers. The findings show that systematic biopsies add limited additional value beyond targeted and ipsilateral systematic biopsies, supporting a targeted and perilesional biopsy approach.

**Clinical Relevance**

Current guideline recommendations for prostate biopsy favor targeted and perilesional sampling without systematic biopsy cores. It is unknown if this biopsy scheme can or should also be applied to other prostate biopsy indications such as biopsies inside of active surveillance protocols. This study shows that systematic biopsy cores, in addition to targeted and perilesional cores, add little additional value when it comes to upgrading as an endpoint. In general only 2–7% of the upgraded tumors were upgraded based on the result of systematic biopsies only. Editor-in-Chief: Jochen Walz.

**Patient Summary**

We studied whether men with prostate cancer on active surveillance need additional random biopsies in addition to biopsies targeted at a suspicious area on magnetic resonance imaging (MRI). We found that targeted biopsies, together with samples taken from the same side as the MRI lesion, detected nearly all cases of more aggressive disease. These findings suggest that a biopsy strategy combining targeted and nearby sampling is sufficient.

## 1 Introduction

Active surveillance (AS) has become a widely accepted management strategy for men with low-risk and selected cases of intermediate-risk prostate cancer (PCa) [1], aiming to delay or avoid unnecessary treatment-related side effects while ensuring timely intervention in case of disease progression. A key element of AS protocols is the use of protocol-driven repeat biopsies to detect any upgrading to or initially missed diagnosis of clinically significant PCa (csPCa) that may require treatment. Nearly all AS protocols recommend repeat biopsies, typically ranging from annually to once every 3 yr [2].

With the increasing use of prostate magnetic resonance imaging (MRI), targeted biopsies (TBx) combined with perilesional biopsies have been shown to reliably detect csPCa in the primary diagnostic setting [3], [4] and during AS [5], leading to European Association of Urology (EAU) guideline recommendations to omit systematic biopsies (SBx) in men with a positive MRI [6]. However, despite these recommendations, SBx is still frequently performed in routine clinical practice and continues to be recommended in some national guidelines [7], [8], [9]. Moreover, much of the existing evidence comes from high-volume expert centers, which may not reflect outcomes in everyday clinical settings.

Therefore, in this study, we investigate the added value of SBx during repeat biopsies in men undergoing AS with a positive MRI at the time of repeat biopsy, following an initial diagnostic MRI, using real-world data from the Prostate Cancer Research International Active Surveillance (PRIAS) study.

## 2 Patients and methods

### 2.1 Study population and study design

We used data from the global PRIAS database [www.prias-project.org]. Since 2020, PRIAS inclusion criteria for patients diagnosed with MRI have been as follows: clinical stage ≤T2, prostate-specific antigen (PSA) ≤20 ng/ml, PSA density ≤0.25 ng/ml^2^, and International Society of Urological Pathology (ISUP) grade group (GG) ≤2 without the presence of cribriform growth (CR) or intraductal carcinoma (IDC) in ≤50% of biopsy cores. Follow-up is scheduled according to a fixed protocol, including PSA testing every 3 mo during the first 2 yr and every 6 mo thereafter, digital rectal examination every 6 mo during the first 2 yr and annually thereafter, and MRI with biopsy at years 1, 4, 7, and 10, and every 5 yr thereafter (Supplementary Fig. 1). In clinical practice, protocol deviations may occur based on clinical judgment.

For this study, we included all repeat biopsies from men diagnosed with PCa who underwent MRI and SBx at diagnosis, with or without TBx depending on the presence of an MRI lesion, had a positive MRI at the time of repeat biopsy, and underwent both SBx and TBx during that procedure. To ensure a uniform baseline diagnostic pathway and avoid introducing differences in initial disease classification, men diagnosed with only TBx were excluded. MRI acquisition and interpretation were performed according to local practice at each participating center. MRI lesions were scored according to the Prostate Imaging Reporting and Data System (PI-RADS) version 1 or 2 guidelines [10] (depending on the time of reporting), with scores of 3–5 considered suspicious for csPCa.

### 2.2 Outcomes and statistical analysis

Histologic upgrading was assessed using three different definitions of upgrading: any increase in ISUP GG compared to the diagnostic biopsy result; upgrading to ISUP GG2 with CR/IDC (GG2+) or higher, reflecting an additional pathological risk stratification within GG2 [11], [12]; and upgrading to ISUP GG≥3, a more general threshold typically triggering a recommendation for treatment [2]. Upgrading was categorized as detected by both SBx and TBx, by TBx only, or by SBx only. Classification was based on whether each biopsy method independently identified the upgrading rather than on the highest GG detected. Therefore, if both SBx and TBx detected upgrading, but one method identified a higher GG, the case was still classified as detected by both SBx and TBx because either method alone would have led to the same clinical management. For upgrading cases to ≥GG2+ detected by SBx only, pathology reports were reviewed to determine laterality relative to the MRI lesion (ie, ipsilateral or contralateral). Instances where MRI lesions were located in the midline were excluded from this analysis, as laterality could not be reliably determined in these cases.

Descriptive statistics were used to report clinical patient characteristics. The proportion of upgrading was reported with corresponding 95% confidence intervals (CI), calculated using the Wilson method. We calculated cumulative incidences of clinically significant upgrading to ≥GG2+ over time, using detection by TBx only, SBx only, and both as mutually exclusive competing events. Follow-up was calculated from the date of diagnosis to the repeat biopsy at which upgrading was detected. Patients without upgrading were censored at the date of their last follow-up visit, the date of AS discontinuation, or at 6 yr, whichever came first. Time-varying Cox proportional hazards regression was considered to identify factors associated with clinically significant upgrading (≥GG2+) detected exclusively by SBx. However, given the limited number of events (*n* = 19), this was considered unreliable and was therefore not performed. All statistical analyses were performed in R version 4.3.3 (R Foundation for Statistical Computing, Vienna, Austria).

## 3 Results

A total of 2316 patients were diagnosed with MRI and SBx (± TBx). After exclusion of patients diagnosed with TBx only (*n* = 49), 2267 patients remained, of whom 1181 underwent at least one repeat biopsy. Of this group, 568 patients from 38 centers across 11 countries underwent at least one repeat biopsy including both SBx and TBx. In total, these patients underwent 650 repeat biopsies with SBx and TBx, making up the dataset for this study (Fig. 1).

**Fig. 1:**
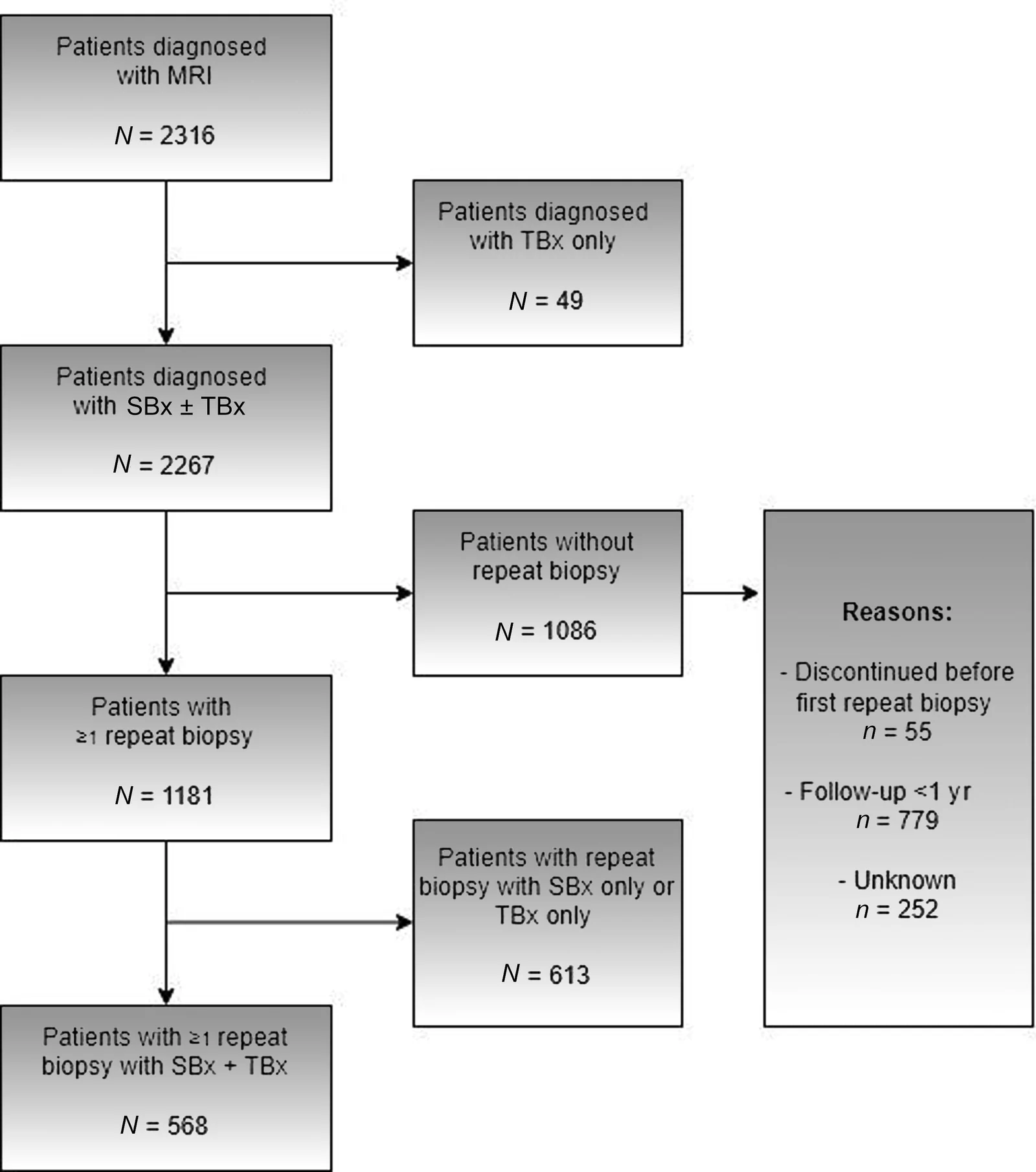
Cohort selection. MRI = magnetic resonance imaging; SBx = systematic biopsies; TBx = targeted biopsies.

At diagnosis, 10% of patients had ISUP GG2 disease. Most lesions were classified as PI-RADS 4, both at diagnosis and on repeat biopsies. Most repeat biopsies represented the patients’ first biopsy after diagnosis and were performed at a median of 1.1 yr later (interquartile range [IQR] = 1.0–1.2; Table 1). The median follow-up of those still on AS was 3.0 yr (IQR = 1.8–4.3), with 88 men already having reached at least 5 yr of AS. CR/IDC status was available for 633 of the 650 repeat biopsies (97%), which were included in the CR/IDC-specific analyses.

**Table 1:** Baseline characteristics

**Characteristic**	**Patients (*N*** = **568)**
**Diagnosis**	
Median (IQR)	
Age (yr)	66 (61–71)
PSA (ng/ml)	6.0 (4.6–7.9)
PSA density (ng/ml/ml)	0.13 (0.09–0.17)
Total cores taken	13 (11–14)
Positive cores	2 (1–3)
*N* (%)	
Clinical stage	
T1c	497 (88)
T2	71 (12)
PI-RADS score	
1–2	123 (22)
3	91 (16)
4	288 (51)
5	62 (11)
Unknown	4
ISUP GG	
1	513 (90)
2	55 (10)
**Repeat biopsies**	**Biopsies (*N*** = **650)**
Median (IQR)	
PSA (ng/ml)	6.9 (4.7–9.5)
PSA density (ng/ml/ml)	0.13 (0.09–0.19)
Total cores taken	12 (10–12)
Positive cores	2 (0–3)
Time between diagnosis and biopsy round (yr)	
First	1.1 (1.0–1.2)
Second	3.1 (2.1–4.1)
Third	5.1 (3.6–6.6)
Fourth	7.9 (6.4–9.3)
Fifth	8.5 (7.2–9.9)
*N* (%)	
PI-RADS score	
3	113 (21)
4	308 (59)
5	105 (20)
Unknown	124
Biopsy round	
First	493 (76)
Second	127 (20)
Third	25 (3.8)
Fourth	3 (0.5)
Fifth	2 (0.3)

IQR = interquartile range; ISUP GG = International Society of Urological Pathology grade group; PI-RADS = Prostate Imaging - Reporting and Data System; PSA = prostate-specific antigen.

### 3.1 Upgrading

The proportion of upgrading stratified by detection method is summarized in Table 2. Any upgrading occurred in 238 of the 650 repeat biopsies (37%; 95% CI = 33–40), of which 47 (7.2%; 95% CI = 5.5–9.5) were detected only by SBx. Among the 633 biopsies with available CR/IDC data, upgrading to ≥GG2+ occurred in 100 (16%; 95% CI = 13–19), of which 19 were identified by SBx only (3.0%; 95% CI = 1.9–4.6). Upgrading to ≥GG3 occurred in 78 of the 650 repeat biopsies (12%; 95% CI = 10–15), of which 15 were identified by SBx only (2.3%; 95% CI = 1.4–3.8%). These numbers translate into a “number needed to systematically biopsy” of 14, 33, and 43, respectively, to detect one additional case of upgrading.

**Table 2:** Upgrading and detection method for each of the three definitions of upgrading

Upgrading definition	Upgrading *n/N* (%; 95% CI)	SBx + TBx *n/N* (%; 95% CI)	TBx only *n/N* (%; 95% CI)	SBx only *n/N* (%; 95% CI)
Any upgrading	238/650 (37; 33–40)	101/650 (16; 13–19)	90/650 (14; 11–17)	47/650 (7.2; 5.5–9.5)
≥GG2+	100/633 (16; 13–19)	34/633 (5.4; 3.9–7.4)	47/633 (7.4; 5.6–9.7)	19/633 (3.0; 1.9–4.6)
≥GG3	78/650 (12; 10–15)	25/650 (3.8; 2.6–5.6)	38/650 (5.8; 4.3–7.9)	15/650 (2.3; 1.4–3.8)

CI = confidence interval; GG = grade group; SBx = systematic biopsy; TBx = targeted biopsy.

Because some patients underwent multiple repeat biopsy sessions during follow-up, the number of biopsy sessions exceeds the number of included patients.

### 3.2 Laterality

Of the 19 cases upgrading to ≥GG2+ found by SBx only, one had an MRI lesion in the midline of the prostate, and one had no information on laterality in the pathology report, leaving 17 cases to analyze. Of these 17 cases, 14 were found ipsilateral to the MRI lesion (82%). In total, contralateral SBx identified upgrading that targeted or ipsilateral SBx did not find in three of the 650 biopsies (0.5%).

### 3.3 Upgrading over time

At 6 yr, the cumulative probability of upgrading to ≥GG2+ was 27% (95% CI = 21–32) compared with 5.4% (95% CI = 2.9–9.1) for upgrading detected exclusively by SBx. The cumulative incidence of upgrading detected by both SBx and TBx and by TBx only were 8.3% (95% CI = 5.4–12) and 13% (95% CI = 9.4–17), respectively (Fig. 2).

**Fig. 2:**
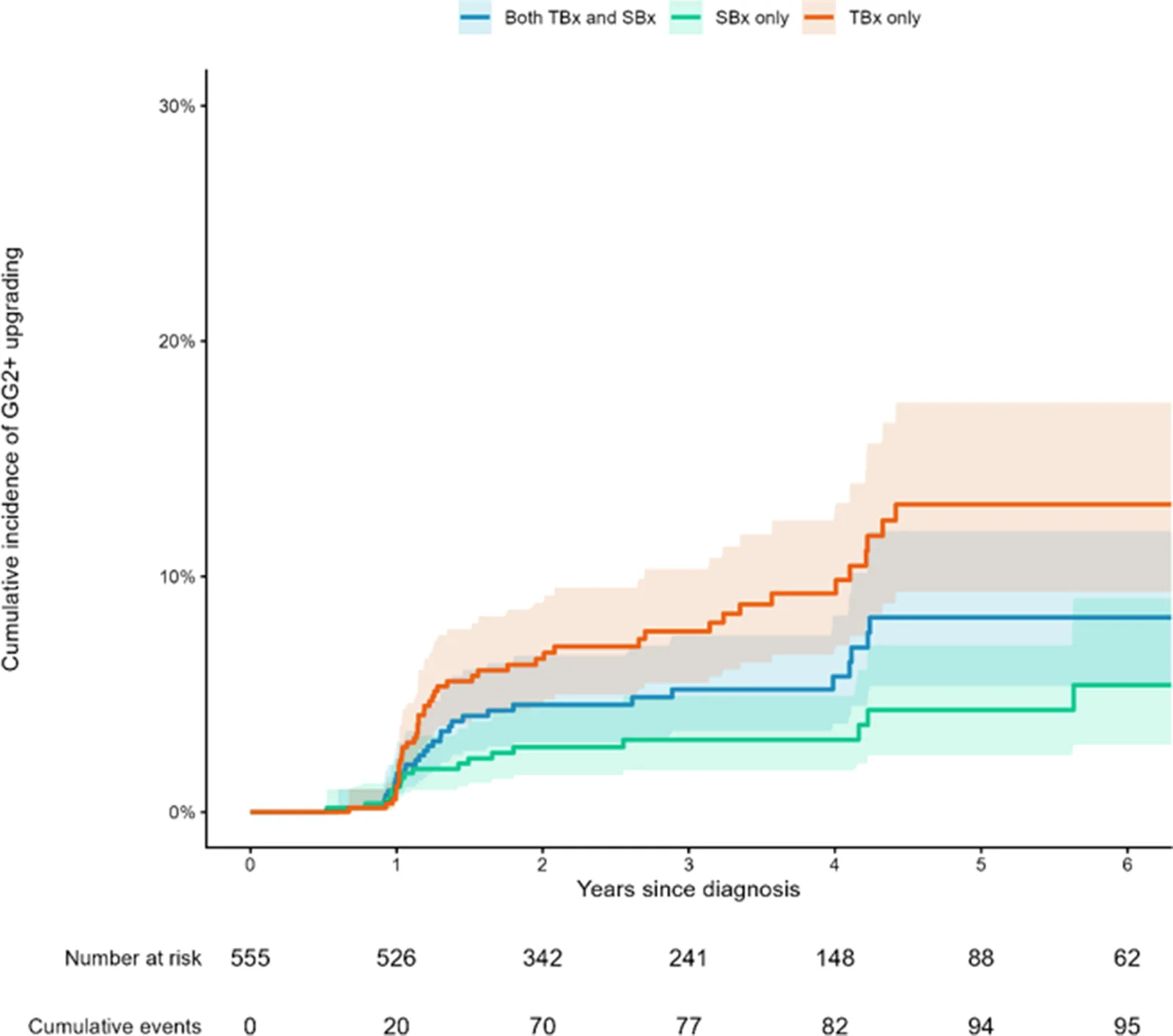
Cumulative incidence of upgrading to ≥GG2+ during active surveillance according to biopsy method of detection. GG = grade group; TBx = targeted biopsies; SBx = systematic biopsies.

## 4 Discussion

In this study, we evaluated the added value of SBx during repeat biopsies in men on AS with a visible lesion on MRI. Using real-world data from the global PRIAS study, we found that SBx detected upgrading that would have been missed by TBx in 7.2% of the biopsies, decreasing to 3.0% when considering clinically relevant upgrading (≥GG2+). This corresponds to a number needed to systematically biopsy of 33 to detect one extra case of upgrading to ≥GG2+. These findings suggest that the overall added value of SBx is limited. Further analysis of the SBx-only detected upgrading cases showed that most (82%) were located ipsilateral to the MRI lesion, whereas upgrading detected exclusively by contralateral SBx was rare (0.5% of the biopsies). This indicates that the (already limited) additional yield of SBx largely comes from tissue contiguous to the lesion rather than from a new tumor focus and likely reflects sampling of the perilesional region rather than systematic sampling across the prostate. Consequently, these findings support a biopsy strategy that combines targeted and perilesional sampling, in line with current EAU guideline recommendations.

Our findings align with those of Fakunle et al [5], who analyzed 821 men on AS (18% with ISUP GG2) and reported that only 5% harbored higher-grade cancer outside the targeted lesion, and only 2% showed aggressive pathologic features. They also showed that 25% of the patients had the dominant lesion in contiguous sites, suggesting that additional sampling of the perilesional area improves detection. In contrast, Osses et al [13] reported a higher added value of SBx in a cohort of 111 men undergoing their first repeat biopsy, with SBx detecting clinically relevant upgrading in 11% of biopsies (compared with 4.8% detected by TBx alone). Nearly half of these upgrades were located contralateral to the MRI lesion. These discrepancies could partly be explained by their smaller sample size (63 biopsies following a positive MRI vs 650 in our study), and ongoing improvements in TBx techniques (eg, software and biopsy route), MRI quality, and increased urologist and radiologist experience, given the rapid developments in these areas in recent years [14], [15], [16]. Contrary to these numbers, studies in biopsy-naive patients have consistently shown that contralateral SBx contributes little, with detection rates ranging from 1.0% to 2.8%, further supporting our findings [17], [18], [19], [20].

In addition, much of the upgrading observed during AS may reflect initial biopsy sampling error rather than actual tumor progression, meaning that taking more biopsy cores may increase upgrading rates without necessarily identifying more clinically meaningful or biologically aggressive disease [21]. This is consistent with our finding that most SBx-only detected upgrades were located ipsilateral to the MRI lesion. Importantly, even if csPCa is missed during repeat biopsy, patients remain under close surveillance, including regular PSA testing and repeat MRI and/or biopsy triggered by a significant PSA rise [2]. Previous studies have shown that a delay of up to 1 yr in detecting csPCa is unlikely to adversely affect oncological outcomes [22], [23], [24], further supporting the safety of a less intensive biopsy approach.

Omitting (contralateral) SBx may reduce patient burden and health care resource use. TBx typically requires 3–5 cores, whereas SBx adds ∼12 additional cores depending on prostate volume [6]. This could result in shorter procedure times, lower costs [25], reduced biopsy-related complications such as infection and pain [26], and potentially improved adherence to AS protocols [27], [28]. In addition, it may decrease the detection of MRI-invisible low-volume Gleason pattern 4 without CR/IDC, which can still be followed up by AS. However, the omission of SBx during AS should be interpreted in a clinical context. In selected clinical scenarios, such as persistently increasing PSA despite stable imaging or discordant clinical findings, SBx may still provide additional value. Furthermore, SBx may add diagnostic information considered relevant for treatment planning, particularly in the context of nerve-sparing surgery and focal therapy, in which detection of contralateral and/or MRI-invisible csPCa may influence patient selection [6], [29]. However, importantly, recent evidence suggests that contralateral SBx has limited impact on surgical planning [30], and that TBx with perilesional sampling may be sufficient for adequate lesion assessment in focal therapy evaluation [31]. From a patient perspective, the benefit of reducing the number of biopsy cores may also be relatively modest when a repeat biopsy is required regardless, and some patients may prefer more extensive sampling if this reduces the risk of missing clinically significant disease. Therefore, the decision to omit SBx should be individualized and incorporate patient preferences.

To our knowledge, this is the first study to evaluate the added value of SBx during AS using a large, real-world, international cohort. Importantly, previous studies have been limited by smaller sample sizes, single-center designs, or more selected populations, whereas our cohort reflects contemporary practice across 11 countries and 38 centers. By including centers with varying levels of MRI quality, radiologist and urologist experience, and biopsy routes and techniques, our findings provide insights into everyday clinical practice, thereby enhancing generalizability.

However, several limitations should be acknowledged. All patients in our cohort underwent SBx at diagnosis, which may limit generalizability to contemporary TBx-only pathways. Furthermore, the retrospective design introduces the possibility of selection and registration bias, potentially influencing the observed upgrading proportions. On the one hand, upgrading may be overestimated, as patients with favorable clinical parameters who omitted a repeat biopsy were not included. On the other hand, upgrading may be underestimated because of the exclusion of patients who underwent repeat biopsy with TBx only, which may have resulted in missed detection of upgrading that would otherwise have been detected by SBx. In addition, a substantial proportion of patients (*n* = 1086) were excluded from analysis as they did not undergo repeat biopsy during follow-up, and the reason could not always be retrieved (*n* = 252), introducing additional potential bias. Furthermore, information on biopsy indication (mandated/for cause), protocol adherence, and biopsy protocol was not systematically recorded, limiting the ability to assess heterogeneity in biopsy practice. Finally, because of the low number of events, we were unable to reliably assess factors associated with clinically significant upgrading detected by SBx, limiting our ability to identify subgroups of patients who may benefit from additional systematic sampling.

Future research should focus on the outcomes of men diagnosed with TBx only, as this population increasingly reflects contemporary clinical practice and may have a different risk profile from cohorts diagnosed using SBx with or without TBx. In particular, the optimal eligibility criteria for AS in the TBx era should be reevaluated, including how tumor volume should be assessed, as traditional measures such as the number of positive biopsy cores are less informative in this context. Furthermore, the relatively low numbers of clinically significant upgrading events in our cohort highlight the need for risk-adapted surveillance strategies that may further reduce unnecessary biopsies without compromising oncological safety.

## 5 Conclusions

The added value of SBx in addition to targeted cores at repeat biopsy during AS appears limited in men with a positive MRI, particularly for contralateral sampling. The number of upgrading cases was low, and most additional upgrading cases detected by SBx were located ipsilateral to the lesion, suggesting that targeted plus perilesional biopsies would capture this added value. These findings support a more selective biopsy approach, limiting the burden of AS follow-up.

  ***Author contributions***: Jeroen J. Lodder had full access to all the data in the study and takes responsibility for the integrity of the data and the accuracy of the data analysis.

  *Study concept and design:* Lodder.

*Acquisition of data*: Marenghi, van den Bergh, Lozano-Uruñuele, Graefen, Rannikko, Staerman, Sugimoto, Tully, Papavassilis, Somford, Roobol, the PRIAS consortium.

*Analysis and interpretation of data*: Lodder, Remmers, van den Bergh, Roobol.

*Drafting of the manuscript*: Lodder.

*Critical revision of the manuscript for important intellectual content*: Lodder, Remmers, Marenghi, van den Bergh, Lozano-Uruñuele, Graefen, Rannikko, Staerman, Sugimoto, Tully, Papavassilis, Somford, Roobol.

*Statistical analysis*: Lodder, Remmers.

*Obtaining funding*: None.

*Administrative, technical, or material support*: None.

*Supervision*: van den Bergh, Roobol.

*Other*: None.

  ***Financial disclosures:*** Jeroen J. Lodder certifies that all conflicts of interest, including specific financial interests and relationships and affiliations relevant to the subject matter or materials discussed in the manuscript (eg, employment/affiliation, grants or funding, consultancies, honoraria, stock ownership or options, expert testimony, royalties, or patents filed, received, or pending), are the following: None.

  ***Funding/Support and role of the sponsor*:** None.

  ***Acknowledgments:*** The authors are grateful to Fabio Badenchini, Conja Franken-Raab, Francisco Lozano-Uruñuela, Pablo Martinez, Ryuji Matsumoto, Philipp Papavassilis, Diederik Somford, and Julio Téigel-Tobar for providing the pathology reports of their patients, enabling us to perform the laterality analysis.

  ***Declaration of generative AI and AI-assisted technologies in the writing process:*** During the preparation of this work, the author used Microsoft Copilot to assist with language refinement and improving clarity of phrasing. After using this tool, the author carefully reviewed, edited, and verified the content as needed and takes full responsibility for the content of the publication.

## Appendix A Supplementary data

Supplementary Data 1 PRIAS consortium.
