# Supplementary material for: Can We Omit Systematic Biopsies in Men with a Positive MRI During Repeat Biopsies for Active Surveillance for Prostate Cancer? Results from the PRIAS Study
Source: Eur Urol Open Sci. 2026 Oct 13;94:31–8. doi: 10.1016/j.euros.2026.09.006 (PMC13683850; doi:10.1016/j.euros.2026.09.006)
Supplement: Supplementary file 1 [file mmc1.docx]

**Appendix A.**

**Members of the PRIAS consortium**

| PRIAS Centre | Name representative(s) |
| --- | --- |
| Admiraal de Ruyter Ziekenhuis te Goes/Vlissingen | E. Planken; J. Jaspars |
| Aker Sykehus, Norway | V. Berge; O.A. Hopland |
| Albert Schweitzer Ziekenhuis | R. Raaijmakers |
| Alysis Zorggroep Arnhem | G. Smits |
| Amphia Ziekenhuis | J. van Brakel; D. van der Schoot |
| Akita University Hospital | T. Habuchi |
| Antoni van Leeuwenhoek Ziekenhuis (NKI-AVL) | H. van der Poel |
| Atrium MC Heerlen | P. de Vries |
| BC Cancer Agency | A. Clarke |
| Bronovo Ziekenhuis | M. Kummeling |
| Canisius-Wilhelmina Ziekenhuis Nijmegen | D.M. Somford; Siebren Dijkstra; Joost de Baaijt |
| Catharina Ziekenhuis Eindhoven | W. Scheepens |
| Centre Hospitalitier Universitaire de Lille | A. Villers |
| Charles University Prague | O. Capoun |
| Chiba University Hospital | T. Ichikawa; S. Sakamoto |
| Chu de Bordeaux | R. Gregoir |
| Diaconesses - Croix St Simon Hospital | B. Guillonneau |
| Diakonessenhuis Utrecht | K. van Dalen |
| Emco Klinik, Salzburg | A. Jungwirth |
| Erasmus MC | M.J. Roobol; C.H. Bangma |
| FCPorto | N. Azevedo |
| FUCDIM | T. Dellavedova |
| Fundaci | F. Sanguedolce |
| Fundación Hospital de Jove | P. Pellejero Pérez |
| G. Fornaroli hospital, Legnano | M. Maffezzini |
| Gelderse Vallei Ziekenhuis, Ede | M. Kortleve |
| GSMS - Kumamoto University | M. Eto |
| Gunma University | K. Suzuki |
| HAGA ziekenhuis | H. Roshani |
| Hamamatsu University Hospital | T. Sugiyama |
| Hara-Sanshin Hospital | K. Shiga |
| Heinrich Heine University of Dusseldorf | D. Caasen-Findeisen |
| HELIOS Klinikum Krefeld | J. Schmitges |
| Hiroshima University Hospital | J. Teishima |
| Hokkaido Cancer Center | Satoru Maruyama |
| Hospital Clinico San Carlos | I. Parra |
| Hospital Italiano de Buenos Aires | P. Francisco Martinez |
| Hospital Universitari Mutua Terrassa | J.M. Caballero Giné; R Bastus |
| Hospital Universitario Marques de Valdecilla | E. Ramos Barselo |
| Hospital San Agustin | F.J. Pérez García |
| Hospital Universitario de Navarra, Pamplona | Francisco Lozano-Uruñuela; M. Montesino |
| HYKS (Helsinki) | A. Rannikko |
| IJsselland Ziekenhuis | B. Rikken |
| Iwate university | W. Obara |
| Jeroen Bosch Ziekenhuis | R.A. Schipper |
| Jikei Medical University Hospital | H. Sasaki |
| Kagawa University | Y. Kakehi |
| Kagawa University | T. Kato; M. Sugimoto |
| Kansai Medical University Hospital | T. Matsuda; H. Kinoshita |
| Kitasato University | K. Tabata |
| Klinik für Urologie Nagold | M. Schiffer |
| Klinikum Aschaffenburg-Alzenau | J. Winkle |
| Kocaeli University Faculty of Medicine, Turkey | T.A. Ozkan |
| Krankenhaus der Barm. Brueder | N. Schmeller |
| Kuopio University Hospital | S. Aaltomaa |
| Kurashiki Medical Center | Y. Yamamoto; A. Terai |
| Kuusankoski Hospital | M. Multanen |
| Kyorin University | H. Fukuhara |
| Kyoto Prefectual University of Medicine | O. Ukimura |
| Kyoto University Hospital | T. Inoue |
| Kyushu University Hospital | A. Yokomizo |
| La Fe University Hospital, Valencia | J.M. Alapont-Alacreu |
| LUMC Leiden | R. Pelger; E. van Gennep |
| Maasstad Ziekenhuis | S. Klaver |
| Marien Hospital Herne | K. Tully |
| Martini-Clinic | M. Graefen; S. Knipper |
| MCMCC - Warsaw | R. Sosnowski |
| Meander MC Amersfoort | M. Schotman |
| Medisch Centrum Alkmaar | J. Verlind |
| Medisch Spectrum Twente | B. Santerse; M.J. Pit |
| Mikkeli Hospital | N. Hendolin |
| Miyazaki University Hospital | N. Terada |
| Nagasaki University graduate school of medicine | T. Hakariya |
| National Hospital Organization Kyoto Medical Cente | H. Okuno |
| Ng Teng Fong General Hospital | L.W. Tim |
| Niigata Cancer Center | Y. Kitamura |
| Niigata University | Y. Tomita |
| Zuyderland Hospital | R. Bosshardt |
| Osaka - Center for Adult Disease and Cancer | M. Nakayama |
| Osaka University Graduate School of Medicine | M. Uemura |
| Oulu University Hospital | M. Vaarala |
| Päijät-Häme Hospital | I. Taina |
| Polycliniques les Bleuets | F. Staerman |
| Praxisgemeinschaft fur Onkologie und Urologie | Dr. Gerald Rodemer |
| Prince of Wales Hospital, Hong Kong | C.F. Ng; P. Chiu |
| Prostatazentrum Elbe-Weser | S. Laabs |
| Reinier de Graaf Gasthuis | J. Hoeven |
| Rode Kruis Ziekenhuis, Beverwijk | N. Bosch |
| Ryukyu University Hospital | S. Saito |
| Saitama Cancer Center | Y. Sakai |
| Sapporo Medical University Hospital | K. Hashimoto |
| Seinäjoki Hospital | M. Leskinen |
| Shikoku Cancer Center | K. Hashine |
| SIURO - ASST Bergamo Est | A. Paganelli |
| SIURO - Azienda Ospedaliera Careggi | A. Lapini |
| SIURO - Azienda Sanitaria dell’Alto Adige | E. Trenti |
| SIURO - Azienda Sanitaria Giovanni XXIII | M. Roscigno |
| SIURO - Desenzano del Garda General Hospital | M. Tanello |
| SIURO - Fondazione IRCCS Istituto | F. Badenchini; C. Marenghi |
| SIURO - Fondazione IRCCS Istituto | M.A. Alvisi |
| SIURO - Istituto humanitas | N. Lo Iacono |
| SIURO - Istituto regina elena | G. Simone |
| SIURO - Ospedale di Trento | O. Caffo |
| SIURO - Ospedale M. Bufalini | G. Cicchetti |
| SIURO - Ospedale sant` anna | G. Conti |
| SIURO - Ospedale umberto i | R. Sanseverino |
| SIURO - Policlinico di Bari | P. Ditonno |
| SIURO - Policlinico sant` orsola | G. Martorana |
| SIURO - Università di Salerno | P. Verze |
| Slingeland Ziekenhuis | A. Geboers |
| Spaarne Ziekenhuis Hoofddorp | A.N. Noordzij |
| St. Anna Ziekenhuis te Geldrop | A. Sonneveld |
| St. Antonius Ziekenhuis | J. Beck; S. van Selm |
| St. Franciscus Gasthuis | E. Boevé; M.B. Busstra; D. van Diepen; R.C.N. van den Bergh |
| St. Jans Gasthuis (Weert) | M. Landman |
| St. Marien Krankenhaus Ahaus-Vreden Gmbh | E. Adrian Treiyer |
| St. Savvas Hospital | T. Anagnostou |
| St.-Vincenz Krankenhaus | M. Gillich |
| Stiftungsklinikum PROSELIS | J. Hanske |
| Tampere University Hospital | T.Tammela |
| Teikyo University Chiba Medical Center | Y. Naya |
| Tergooiziekenhuizen | F. van der Windt |
| Tochigi Cancer Center | K. Kawashima |
| Toho University Sakura Hospital | N. Kamiya |
| Tohoku University Hospital | K. Mitsuka |
| Tokai University Hospital | T. Nomoto |
| Tokushima University Hospital | Y.Kusuhara |
| Tokyo Kosei Nenkin Hospital | K. Akakura |
| Tokyo Womens` Medical University | J. Iizuka |
| Tweesteden ZKH Tilburg | B. Wijsman |
| UMC St. Radboud Nijmegen | I. van Oort |
| Univ. Hospital Muenster | P. Papavassilis |
| Univ. Hospital Muenster | A. Semjonow |
| Université Jean Monnet, St Etienne | N. Mottet |
| University Hospital A Coruna | F. Gomez Veiga |
| University Hospital Malmö | A. Bjartell |
| University Hospital Príncipe de Asturias | J. Serrano Uribe; J. Téigell-Tobar |
| University of Tokyo | H. Fukuhara; M. Nakamura |
| Urologia Marina Alta | T. Keul |
| Urologie Mönchengladbach | J. Schmitges |
| Urologische Gemeinschaftspraxis Buxtehude | H. Hollberg; T. Dill; I. Kaspers; Z. Türk |
| Urologische GP Pruener Gang Kiel | J. Lehmann |
| Urologische Klinik Sindelfingen | M. Ruhs |
| USANZ - Ascot Hospital | C. Hawke |
| USANZ - Auckland Hospital, Private Practice | M. Rice |
| USANZ - Austin and The Northern Hospital | D. Gyomber |
| USANZ - Austin Hospital | N. Lawrentschuk |
| USANZ - Ballarat Urology | L. Johns-Putra |
| USANZ - Capital Urology Australia P/L | H. Fan Chan |
| USANZ - Central Coast LHD | F. Macneil |
| USANZ - Dr Mark Louie-Johnsun | M. Louie-Johnsun |
| USANZ - Epworth Hospital | P. Anderson |
| USANZ - Geelong Urology | K. Rantall |
| USANZ - Metropolitan Urology | P. Ruljancich |
| USANZ - Nepean Urology Victoria | A. Jayathillake |
| USANZ - Palmerston North Hospital | C. Chemasle |
| USANZ - Perth Urology Clinic | S. La Bianca |
| USANZ - Peter MacCallum Cancer Centre | D. Murphy |
| USANZ - Private Practice Michael J Monsour | M.J. Monsour |
| USANZ - Rockhampton Base Hospital | A. Vega |
| USANZ - St George Hospital | C. Smiles |
| USANZ - St George Private Medical Centre Sydney | D. Malouf |
| USANZ - The Alfred Hospital Melbourne | J. Grummet |
| USANZ - University of Sydney | M. Patel |
| USANZ - University of Sydney | V. Chalasani |
| USANZ - Urology Centre Port Macquarie | P. Rashid |
| USANZ - Urology North | S. Brough |
| USANZ - West Coast Urologists | A. Tan |
| Uwe Behrendt | U. Behrendt |
| Viecuri Medisch Centrum | M. de Bruin-Titulaer |
| VUMC Amsterdam | A.N. Vis |
| Wakayama Prefectural Medical University | I. Hara |
| Westfries Gasthuis | M. Leter |
| Yamagata University Hospital | N. Tsuchiya |
| Yamaguchi University Graduate School of Medicine | H. Matsumoto |
| Zaans Medisch Centrum | I. Proost |
| Ziekenhuis Bernhoven (Veghel) | A.Q.H.J. Niemer |
| Ziekenhuisgroep Twente | E.B. Cornel |

**Appendix B.**

**Supplementary Figure 1. PRIAS follow-up schedule.**

*DRE = digital rectal examination; MRI = magnetic resonance imaging; PSA = prostate specific antigen.*
